# Exploring the Effect of Deep-Sea Water on the Therapeutic Potential of the Anti-Inflammatory Response in an Indomethacin-Induced Gastric Ulcer Rat Model

**DOI:** 10.3390/ijms242417430

**Published:** 2023-12-13

**Authors:** Soo-yeon Park, Jin A Im, Ji Yeon Kim

**Affiliations:** 1 Department of Food Science and Technology, Seoul National University of Science and Technology, Seoul 01811, Republic of Korea; sooyeon.park@seoultech.ac.kr (S.-y.P.); jina51252641@nate.com (J.A.I.); 2 Department of Nano Bio Engineering, Seoul National University of Science and Technology, Seoul 01811, Republic of Korea

**Keywords:** gastric ulcers, deep-sea water, mineral water, indomethacin, anti-inflammatory response, gastroprotection, gastrointestinal health

## Abstract

Gastric ulcers are often exacerbated by factors such as nonsteroidal anti-inflammatory drugs (NSAIDs) and inflammation, and they have a substantial impact on a significant portion of the population. Notably, indomethacin is recognized as a prominent contributor to ulcers. This study investigated this potential method, with normalization to the anti-inflammatory and antiulcer properties of deep-sea water (DSW)-derived mineral water, using an indomethacin-induced gastric ulcer model in rats. The study involved four groups (*n* = 6 rats/group): normal control group (CON), indomethacin-only group (IND), indomethacin with trace mineral water group (TM), and indomethacin with high magnesium low sodium water group (HMLS). For three weeks, the CON and IND groups consumed tap water, while the TM and HMLS groups had access to mineral water. Gastric ulcers were induced on the final day using indomethacin, for all groups except the CON group. The results demonstrated that HMLS intake significantly improved gastric mucosal damage, preserved mucin stability, and increased gastric thickness, indicating its potential to prevent and alleviate indomethacin-induced gastric ulcers. Furthermore, HMLS consumption led to the upregulation of key genes associated with inflammation and a reduction in inflammatory cytokines. These findings suggest that DSW-derived mineral water, and particularly its high Mg^2+^ content, may offer promising health benefits including anti-inflammatory and anti-ulcer properties.

## 1. Introduction

Gastric ulcers are a condition characterized by the erosion of the gastric mucosal layer, or the formation of a cavity on the tissue surface due to damage, typically caused by inflammation [1,2]. Prevalent gastrointestinal disorders develop due to an imbalance between disrupted factors, including *Helicobacter pylori* infections, nonsteroidal anti-inflammatory drugs (NSAIDs), and acid, and protective factors, such as mucin, bicarbonate, and prostaglandins. This imbalance, exacerbated by variables such as stress, alcohol consumption, and prolonged NSAID use, leads to ulcers, which are a recurrent condition with a high prevalence of 5–10% throughout a lifetime [1,3,4,5]. In a comprehensive study of patients diagnosed with gastric ulcers, it was determined that a noteworthy 25% of these cases were attributed to drug-induced ulcers. Notably, NSAIDs, often prescribed for the management of conditions such as arthritis, cardiovascular diseases, colorectal cancer, and Alzheimer’s disease prevention, were the predominant agents responsible for ulcer development, with indomethacin emerging as a prominent example [6,7,8,9]. Indomethacin inhibits the activity of cyclooxygenase (COX) by suppressing the synthesis of prostaglandin. By inhibiting prostaglandin production, indomethacin disrupts the delicate balance between pro-inflammatory and anti-inflammatory mediators. The prolonged use of indomethacin can significantly upregulate the expression of pro-inflammatory cytokines, including tumor necrosis factor-alpha (TNF-α) and interleukin-1β (IL-1β), which are mediated by Th1 cells, potentially leading to inflammation in the gastric mucosa. The increased inflammation and cytokine imbalance induced by indomethacin can ultimately lead to the development of gastric ulcers [6,10,11,12]. To prevent and treat gastric ulcers, traditional healers and herbalists have historically employed natural plant-based remedies. Over the past few decades, there has been a growing focus on scientific research exploring the potential of medicinal plant products for gastroprotection, aiming to identify them as viable therapeutic alternatives [13,14].

Water constitutes a fundamental component in all living organisms, serving diverse functions such as regulating body temperature, enhancing metabolism, and providing essential minerals to the body. Various water sources, including surface water, groundwater, and seawater, are available. In particular, deep-sea water (DSW) has the potential to be a valuable water source due to its capacity to supply essential minerals crucial for health. The minerals contained in DSW can emerge as substantial elements influencing the treatment of diseases, given their pivotal involvement in cellular homeostasis and physiological responses [15,16,17]. DSW refers to seawater found at depths exceeding 200 m; it is characterized by elevated concentrations of minerals such as magnesium (Mg^2+^), calcium (Ca^2+^), potassium (K^+^), and zinc (Zn), along with various nutrients. It is extensively utilized, primarily in the fields of food, agriculture, cosmetics, and medicine, owing to its abundance compared to other natural resources [16,17,18]. In contrast to Ca^2+^, which has demonstrated a high bioavailability in mineral water in most studies, research on the bioavailability of Mg^2+^ is limited. In our previous study, we observed that mineral water with a high Mg^2+^ content derived from DSW alleviated inflammatory symptoms and suppressed inflammatory chemokine genes in dextran sulfate sodium (DSS)-induced enteritis in mouse models [19]. While the precise mechanisms underlying the beneficial effects of Mg^2+^ remain incompletely understood, the results from animal and cell models demonstrate anti-inflammatory properties. Mg^2+^ significantly reduces the levels of pro-inflammatory cytokines, including TNF-a, IL-1β, and IL-6. It plays a role in neutralizing gastric acid and decreasing acid secretion, thus contributing as an appropriate mediator for disease healing. Mg^2+^ supplementation is associated with a reduction in NF-kB activation, leading to decreased production of cytokines and chemokines. Notably, Mg^2+^ stands out as the fourth most prevalent divalent cation in the body [20,21,22]. Serum Mg^2+^ levels are closely regulated within a narrow range by the kidneys and the gastrointestinal tract. Research, including studies involving animal models, suggests that Mg^2+^ deficiency is associated with oxidative stress and an increase in pro-inflammatory cytokine levels that commonly contribute to an inflammatory state [22,23]. Therefore, this study aimed to investigate whether mineral water with a high Mg^2+^ content derived from DSW ameliorates the symptoms of gastric ulcers using an indomethacin-induced model.

## 2. Results

### 2.1. Mineral Composition of DSW-Derived Water Samples

The major mineral contents of DSW and DSW-derived water samples are shown in Table 1. The amount of sodium (Na^+^) contained in the DSW was considerably higher compared to the drinking water generally consumed. Regarding the principal divalent cations in DSW, Ca^2+^ and Mg^2+^, the Mg^2+^ content was approximately 3.54 times higher than that of Ca^2+^. Two samples derived from the DSW were prepared through desalting and the addition of ions. The TM sample demonstrated a Mg^2+^ to Ca^2+^ ratio of 3:1, similar to the DSW with trace minerals. The HMLS sample exhibited elevated Mg^2+^ and Ca^2+^ content consistent with the composition of the DSW.

### 2.2. Effect of DSW-Derived Mineral Water on Gastrointestinal Tissue Weight

The effect of TM and HMLS intake on the weight of gastrointestinal tissue is presented in Table 2. A single oral administration of 50 mg/10 mL/kg B.W. indomethacin was performed in the IND (only), IND + TM, and IND + HMLS groups. The stomach weight of the IND group, in which gastric ulcers were induced, was increased by 15.5% compared to the CON group, and a similar 15.5% increase was observed in the TM. The stomach weight of the HMLS group was increased by 15.04%. No statistically significant differences were observed in any of the groups when compared to the CON group.

### 2.3. Effects of DSW-Derived Mineral Water on IND-Induced Gastrointestinal Mucosal Inflammation Symptoms

**Figure 1:**
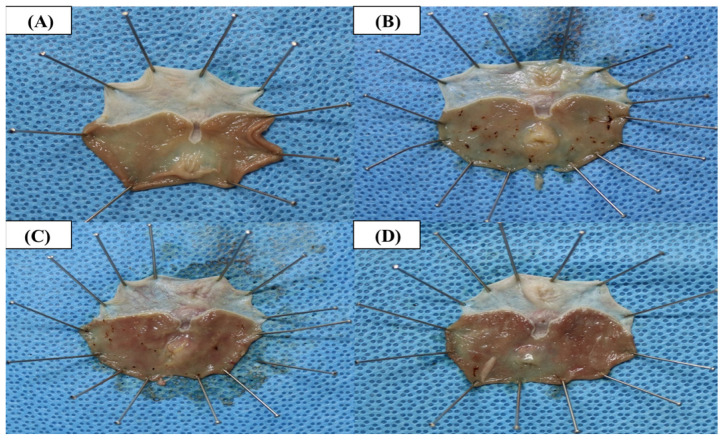
Impact of DSW-derived mineral water on pathological changes in gastrointestinal mucosal inflammation symptoms. (**A**) CON, (**B**) IND, (**C**) TM, (**D**) HMLS. Representative gastrointestinal tissue from each group. CON, normal control (*n* = 6); IND, gastric ulcer induced by indomethacin (50 mg/10 mL/kg B.W. in 0.5% CMC solution) (*n* = 6); TM, gastric ulcer induced by indomethacin with TM (*n* = 6); HMLS, gastric ulcer induced by indomethacin with HMLS (*n* = 6).

Figure 1 presents images of representative gastric tissues from each group, sectioned along the greater curvature. In comparison to that of the CON group, the stomach of the IND group exhibited severe tissue damage and hemorrhagic mucosal lesions. The tissue and mucosal appearance of the HMLS group most closely resembled that of the CON group (Figure 1B,D).

### 2.4. Histological Changes in IND-Induced Gastrointestinal Mucosal Inflammation Caused by DSW-Derived Mineral Water Intake

**Figure 2:**
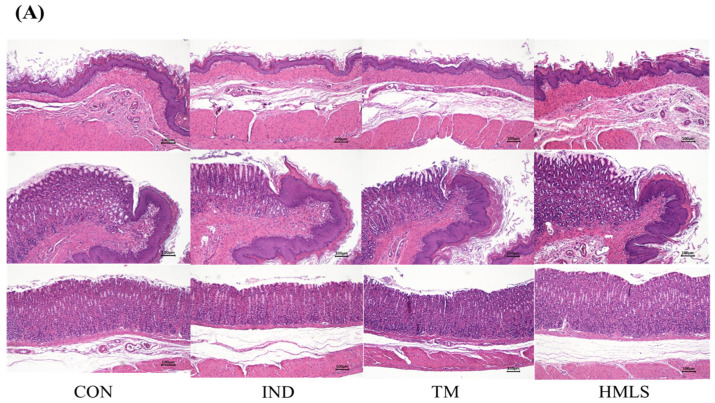

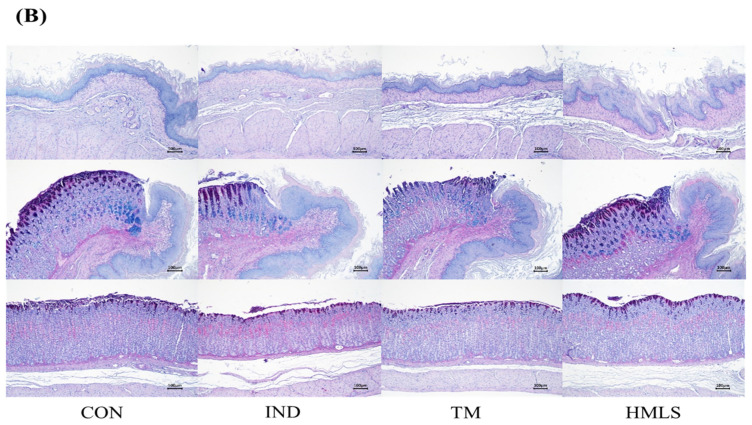
Histological assessment of gastrointestinal mucosal inflammation induced by DSW-derived mineral water in IND-induced gastric ulcer rats. (**A**) H&E staining and (**B**) AB-PAS staining. Representative histopathological images of the gastrointestinal mucosa in Sprague–Dawley (SD) rats with gastric ulcers induced by indomethacin (50 mg/10 mL/kg B.W. in 0.5% CMC solution). Images were captured at 100× magnification (scale bar = 100 µm).

Figure 2A shows the thickness of gastric tissue, as determined by H&E staining, providing a visual representation of stomach thickness in each group. Moreover, the AB-PAS staining in Figure 2B visualizes and identifies the mucin content within the stomach tissues. A measurement of gastric tissue thickness by H&E staining indicated that the IND group exhibited a reduction in gastric thickness compared to both the TM and HMLS groups. Specifically, the IND group displayed a 1.25-fold decrease in gastric thickness in comparison to the CON group. Notably, the HMLS group closely resembled the CON group. Statistically significant differences were detected among the groups. Furthermore, analysis of mucin staining in the gastric mucosal tissue through AB-PAS revealed structural damage to mucin in the IND group when compared to the TM and HMLS groups. Moreover, the mucin conformation appeared more stable in the HMLS group than in the TM group. These findings support that HMLS intake contributed to the mitigation of histopathological symptoms by preserving mucin stability in the gastric mucosal tissue and preventing a decrease in gastric thickness (Figure 2).

### 2.5. Regulation of COX Expression and PGE2 Synthesis in DSW-Derived Mineral Water

**Figure 3:**
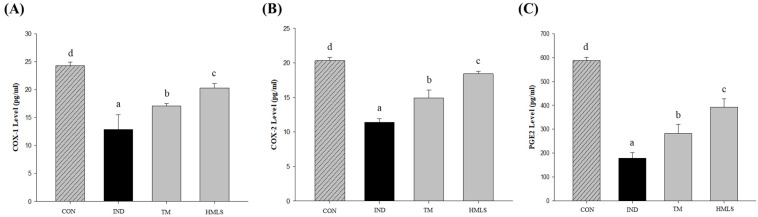
Effect of DSW-derived mineral water on cyclooxygenase (COX) expression and prostaglandin E2 (PGE2) synthesis in indomethacin-induced gastric ulcer rats. (**A**) COX-1 and (**B**) COX-2 expression, and (**C**) PGE2 synthesis in indomethacin (50 mg/10 mL/kg B.W. in 0.5% CMC solution) induced ulcerated rats. Data are presented as the mean ± standard error (S.E.). Different letters above the error bars indicate significant differences at *p* < 0.05 according to an analysis of variance (ANOVA) followed by the Duncan’s multiple comparison test. CON, normal control (*n* = 6); IND, gastric ulcer induced by indomethacin (*n* = 6); TM, gastric ulcer induced by indomethacin with TM (*n* = 6); HMLS, gastric ulcer induced by indomethacin with HMLS (*n* = 6).

IND induces gastric ulcers by inhibiting the production of COX-1, COX-2, and PGE2. COX-1 levels were reduced by 1.89-fold in the IND group compared to the CON group, and the expression of COX-1 in the HMLS group was similar to that of the CON group (Figure 3A; *p* < 0.05). COX-2 levels were decreased by 1.78-fold in the IND group compared to the CON group. Notably, the HMLS group demonstrated COX-2 expression similar to the CON group, with only a 1.10-fold difference observed (Figure 3B; *p* < 0.05). The levels of PGE2 were decreased 3.29-fold in the IND group compared to the CON group, and the difference between the HMLS group and the control group was 1.5-fold. These findings indicate that the expression levels of PGE2 in the CON group and the HMLS group were high (Figure 3C; *p* < 0.05).

### 2.6. Effects of DSW-Derived Mineral Water on MPO Activity and Inflammatory Cytokines Expressed by Th1 and Th2 Lymphocytes

**Figure 4:**
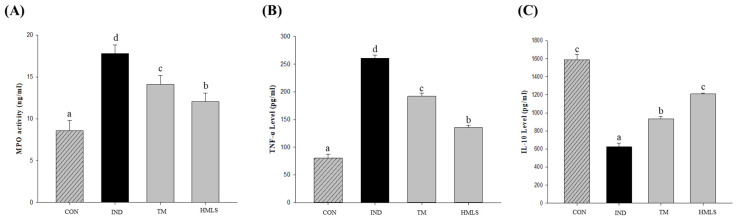
Effects of DSW-derived mineral water on myeloperoxidase activity and inflammatory cytokines in indomethacin-induced gastric ulcer rats. (**A**) Myeloperoxidase (MPO) activity, (**B**) Tumor necrosis factor alpha (TNF-α), and (**C**) Interleukin (IL)-10 levels in indomethacin (50 mg/10 mL/kg B.W. in 0.5% CMC solution) induced ulcerated rats. Data are presented as the mean ± standard error (S.E.). Different letters above the error bars indicate significant differences at *p* < 0.05 according to an analysis of variance (ANOVA) followed by the Duncan’s multiple comparison test. CON, normal control (*n* = 6); IND, gastric ulcer induced by indomethacin (*n* = 6); TM, gastric ulcer induced by indomethacin with TM (*n* = 6); HMLS, gastric ulcer induced by indomethacin with HMLS (*n* = 6).

MPO activity is known to be overexpressed in several inflammatory diseases. It has been reported to increase in ulcerated tissues and decrease in healed tissues. MPO activity serves as a marker for confirming the infiltration of inflammatory cells in gastric tissue. In the IND group, MPO activity was elevated by 2.07-fold compared to the CON group, while the HMLS group exhibited a 1.41-fold increase compared to the control group (Figure 4A; *p* < 0.05). Notably, in the HMLS group, MPO activity was significantly suppressed by IND.

The level of TNF-α increased by 3.23-fold in the IND group compared to the CON group (*p* < 0.05), and this level increased by 1.68-fold in the HMLS group compared to the CON group (*p* < 0.05). Ingestion of HMLS in DSW-derived mineral water was found to reduce the level of TNF-α, an inflammatory cytokine induced by Th1 lymphocytes (Figure 4B).

To assess the effect of DSW-derived mineral water on IL-10, which is an inflammatory cytokine induced by Th2 lymphocytes in response to IND, IL-10 levels were determined using an ELISA kit. The level of IL-10 was reduced by 2.55-fold in the IND group compared to the CON group (*p* < 0.05), and the IL-10 level in the HMLS group decreased by 1.31-fold compared to the CON group (*p* < 0.05). This difference resembles that of the CON group compared to the TM group (Figure 4C). IL-10 is influenced by PGE, and since IND administration suppresses PGE production, the level of IL-10 is also reduced in the IND group due to this effect.

### 2.7. Effects of DSW-Derived Mineral Water on COX and Prostaglandin Synthase (PGES) mRNA Expression

**Figure 5:**
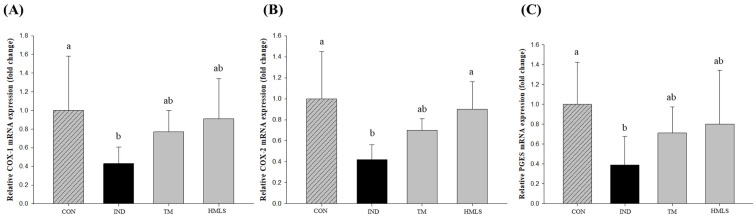
Effect of DSW-derived mineral water on cyclooxygenase (COX) and prostaglandin E synthase (PGES) mRNA expression in indomethacin-induced gastric ulcer rats. (**A**) COX-1, (**B**) COX-2, and (**C**) PGES mRNA expression levels were quantified by RT–qPCR in indomethacin (50 mg/10 mL/kg B.W. in 0.5% CMC solution) induced ulcerated rats. Different letters above the error bars indicate significant differences at *p* < 0.05 according to an analysis of variance (ANOVA) followed by the Duncan’s multiple comparison test. CON, normal control (*n* = 6); IND, gastric ulcer induced by indomethacin (*n* = 6); TM, gastric ulcer induced by indomethacin with TM (*n* = 6); HMLS, gastric ulcer induced by indomethacin with HMLS (*n* = 6).

To elucidate the mechanisms underlying the influence of DSW-derived mineral water on COX-1, COX-2, and PGES expression, the mRNA levels were examined. COX-1 levels experienced a 2.32-fold reduction in the IND group compared to the CON group, with a 1.09-fold decrease observed in the HMLS group relative to the CON group (Figure 5A; *p* < 0.05). Notably, among all of the DSW-derived mineral water samples, the expression in HMLS closely resembled that of the CON group. Similarly, COX-2 levels demonstrated a 2.38-fold decrease in the IND group compared to the CON group, with a 1.11-fold reduction observed in the HMLS group relative to the CON group (Figure 5B; *p* < 0.05). PGES levels experienced a 2.56-fold decrease in the IND group compared to the CON group, with a 1.25-fold reduction observed in the HMLS group relative to the CON group (Figure 5C; *p* < 0.05). Similar to the expression pattern of COX-1, the expression of PGES in HMLS appeared to be similar to that of the CON group among the DSW-derived mineral water samples.

### 2.8. Effects of DSW-Derived Mineral Water on Inflammatory Cytokine mRNA Expression Induced by Th1 and Th2 Lymphocytes

**Figure 6:**
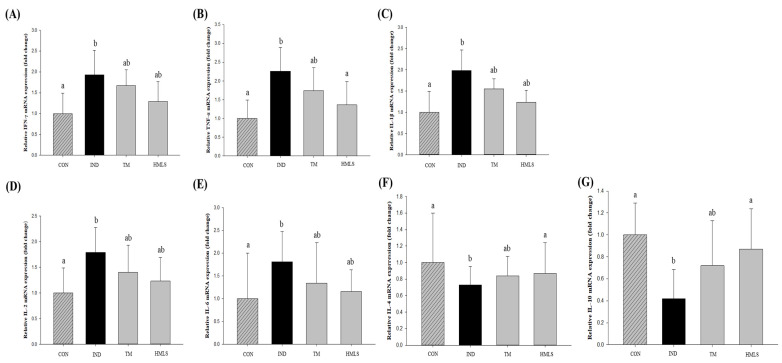
Effects of DSW-derived mineral water on inflammatory cytokine mRNA expression by Th1 and Th2 lymphocytes in indomethacin-induced gastric ulcer rats. (**A**) Interferon gamma (IFN-γ), (**B**) Tumor necrosis factor alpha (TNF-α), (**C**) Interleukin 1 beta (IL-1β), (**D**) Interleukin 2 (IL-2), and (**E**) Interleukin 6 (IL-6) mRNA expression levels for Th1 lymphocytes, and (**F**) Interleukin 4 (IL-4) and (**G**) Interleukin 10 (IL-10) mRNA expression levels for Th2 lymphocytes in indomethacin (50 mg/10 mL/kg B.W. in 0.5% CMC solution) induced ulcerated rats. Different letters above the error bars indicate significant differences at *p* < 0.05 according to an analysis of variance (ANOVA) followed by the Duncan’s multiple comparison test. CON, normal control (*n* = 6); IND, gastric ulcer induced by indomethacin (*n* = 6); TM, gastric ulcer induced by indomethacin with TM (*n* = 6); HMLS, gastric ulcer induced by indomethacin with HMLS (*n* = 6).

The inflammatory cytokine IFN-γ, produced by Th1 lymphocytes, exhibited a 1.93-fold increase in mRNA expression in the IND-only group compared to the CON group. In the HMLS group, there was a 1.29-fold increase, with expression levels resembling those in the CON group (Figure 6A; *p* < 0.05). TNF-α mRNA expression in the IND-only group showed a significant 2.26-fold increase compared to the CON group, whereas the HMLS group displayed a pattern similar to the CON group (Figure 6B; *p* < 0.05). IL-1β mRNA expression levels were elevated by 1.99-fold in the IND-only group and by 1.24-fold in the HMLS group compared to the CON group (Figure 6C; *p* < 0.05). IL-2 mRNA expression levels in the IND-only group were notably increased by 1.79-fold, and in the HMLS group there was a 1.23-fold increase compared to the CON group (Figure 6D; *p* < 0.05). Additionally, IL-6 mRNA expression levels were elevated by 1.81-fold in the IND-only group and by 1.16-fold in the HMLS group compared to the CON group (Figure 6E; *p* < 0.05).

On the other hand, IL-4, an inflammatory cytokine expressed by Th2 lymphocytes, exhibited decreased mRNA expression levels by 1.37-fold in the IND group compared to the CON group. In the HMLS group, there was a 1.15-fold decrease compared to the CON group (Figure 6F; *p* < 0.05). IL-10 mRNA expression levels showed a significant 2.38-fold decrease in the IND group compared to the CON group, and in the HMLS group there was a 1.15-fold decrease compared to the CON group (Figure 6G; *p* < 0.05).

### 2.9. Total RNA Sequencing and Differentially Expressed Gene Analysis

**Figure 7:**
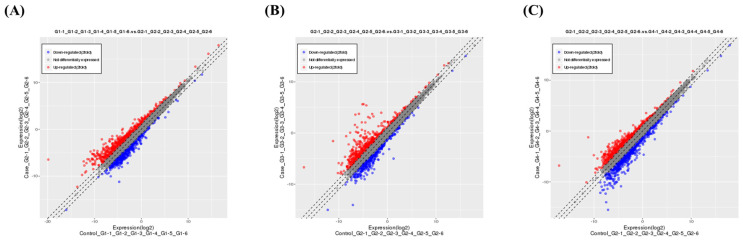
Differentially expressed genes in the ulcer gastric tissue observed in the groups by RNA−seq analysis. Scatter plots for the correlations of mRNA expression levels (log) between groups: (**A**) CON vs. IND, (B) IND vs. TM, and (**C**) IND vs. HMLS. In the scatter plots, red and blue dots represent genes with significantly changed expression, and gray dots represent genes with unchanged expression.

**Figure 8:**
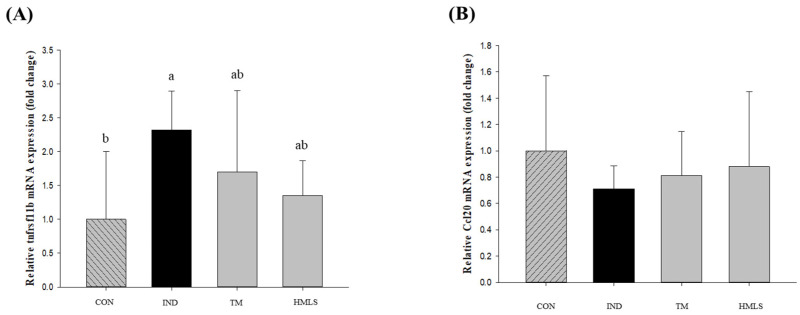
Comparison of the expression levels of genes related to the inflammatory response by RT–qPCR. (**A**) Tnfrsf11b and (**B**) Ccl20. Different letters above the error bars indicate significant differences at *p* < 0.05 according to an analysis of variance (ANOVA) followed by the Duncan’s multiple comparison test. CON, normal control (*n* = 6); IND, gastric ulcer induced by indomethacin (*n* = 6); TM, gastric ulcer induced by indomethacin with TM (*n* = 6); HMLS, gastric ulcer induced by indomethacin with HMLS (*n* = 6).

Differentially expressed genes (DEGs) were determined for gastrointestinal tissue across three comparisons: CON vs. IND, IND vs. TM, and IND vs. HMLS. DEGs were determined by applying Cufflinks software (v2.2.1), with a log2-fold change (FC) and a false discovery rate (FDR) value cutoff of 0.1. A total of 232 DEGs were identified in these comparisons. Of these DEGs, 202 were upregulated and 30 were downregulated in CON vs. IND, 88 were upregulated and 36 were downregulated in IND vs. TM, and 74 were upregulated and 128 were downregulated in IND vs. HMLS. A scatter plot was used to illustrate the fold change (FC) values for all genes concerning the adjusted *p*-value for differential expression in the CON vs. IND, IND vs. TM, and IND vs. HMLS groups (Figure 7). Among the inflammation-related DEGs between the HMLS and IND groups, the expression of the TNF receptor superfamily member 11b (Tnfrsf11b) was notably reduced in the HMLS group compared to the IND group (Figure 8). Moreover, C-C motif chemokine ligand 20 (Ccl20), which plays a crucial role in the immune response by attracting immune cells to sites of inflammation, exhibited the most significant induction, with an FDR value less than 0.1. These findings are summarized in Table 3.

## 3. Discussion

DSW-derived mineral water has been recognized for its various health benefits, including its reported effects on arteriosclerosis and hyperlipidemia, and its antidiabetic, antiobesity, antioxidant, and anticancer effects [15,16,17,24,25]. DSW is attracting significant attention owing to its high productivity, substantial quantity, and potential for energy recycling. The rationale behind selecting DSW for research studies is multifaceted, encompassing its distinct composition and potential health-promoting properties. DSW stands out due to its unique mineral content, notably enriched with essential elements such as Mg^2+^ and Ca^2+^. These minerals play pivotal roles in various physiological processes, and in investigating the impact of DSW on human health [18,26]. However, there has been little research on its potential to alleviate gastric ulcers to date. To our knowledge, this study is the first to investigate the potential mechanism of inflammation underlying the amelioration of gastric ulcer symptoms through RNA sequencing, using mineral-rich DSW-derived mineral water.

In this study, a gastric ulcer model was established using indomethacin, an NSAID commonly associated with mucosal damage in the gastrointestinal tract. This model produced indomethacin-induced gastric mucosal damage and a reduction in gastric thickness, which is consistent with prior research on NSAID-mediated gastric ulcers [11,27,28]. The extent of mucin damage and the reduction in gastric thickness were observed through microscopy [29].

To investigate the impact of DSW-derived mineral water on gastric ulcers, SD rats were provided with tap water containing DSW-derived mineral water ad libitum for 21 days. Notably, there was no significant difference in gastric weight due to ulceration between the groups. Nevertheless, an improvement in gastric mucosal damage and an increase in gastric thickness were observed in the HMLS group. These results strongly support the notion that Mg^2+^, a crucial component of DSW-derived mineral water, holds the potential to prevent ulcer formation and enhance mucus production, as previously reported [30,31]. Notably, Mg^2+^ has demonstrated anti-inflammatory and ulcer-preventing properties [2,32]. In particular, HMLS, characterized by its high Mg^2+^ and low Na^+^ content, demonstrated superior efficacy in promoting gastric health compared to tap water with trace minerals.

Additionally, this study showed that indomethacin had an impact on the levels of various inflammatory cytokines at the mucosal level. The levels of IFN-γ, IL-1β, and IL-2, which are typically induced by Th1 lymphocytes, were reduced, and the levels of IL-6 and TNF-α were significantly increased. Interestingly, indomethacin suppressed the levels of IL-4 and IL-10, which are inflammatory cytokines associated with Th2 lymphocytes. The established role of Mg^2+^ as an anti-inflammatory agent in both animal and cell-based models is noteworthy, as it has been linked to the stimulation of gastric mucus production, protection of the gastric mucosa, reduced acid secretion, and ulcer prevention [2,32,33,34,35]. This study provides evidence that the substantial Mg^2+^ content in HMLS effectively alleviates the symptoms associated with indomethacin-induced gastric ulcers.

Furthermore, it is worth noting that the benefits of Mg^2+^ extend beyond gastrointestinal protection, encompassing its antioxidant properties and potential applications in diabetes and cardiovascular disease management [36,37]. High sodium content in the diet may contribute to the development of hypertension, while Mg^2+^ supplementation has the potential to reduce blood pressure by inhibiting adrenergic activity and reducing vascular tone [38,39,40]. In our study, ulcers induced by indomethacin resulted in a decrease in COX-1 and COX-2 expression levels and reduced PGE synthesis. However, the expression levels of both COX-1 and COX-2 were upregulated and PGE synthesis was increased in the HMLS group. These findings align with previous findings that Mg^2+^ stimulates PGE production while simultaneously reducing gastric acid secretion [35]. This suggests that Mg^2+^ plays a crucial role in providing gastrointestinal protection by inhibiting the action of indomethacin and enhancing PGE production [2,41].

In the RNA-seq analysis of gastric ulcer tissue induced by indomethacin, the DEGs between the IND group and the HMLS group revealed a notable decrease in the expression of tnfrsf11b, an inflammation-related gene. Likewise, the expression levels of TNF-α, a pivotal inflammatory cytokine, were reduced in the HMLS group. In contrast, the expression levels of the immune-related gene Ccl20 were increased in the HMLS group, suggesting a potential amelioration of indomethacin-induced ulcer symptoms. These findings are consistent with previous research indicating that the high Mg^2+^ content in HMLS contributes to anti-inflammatory and ulcer prevention properties [2,32].

In the general population, the primary source of Mg^2+^ intake is the diet, with only a minor portion derived from drinking water. Nevertheless, the consumption of Mg^2+^-rich drinking water has been associated with favorable effects on gastric health and a reduced risk of gastric cancer [42,43]. Notably, Mg^2+^ from water sources is believed to be more readily absorbed than that from dietary sources, underscoring the potential advantages of drinking water with higher Mg^2+^ content [44]. These findings prompt consideration for further research into the intake of both Ca^2+^ and Mg^2+^, encompassing both dietary and water sources, with a focus on human studies. Moreover, rivers and saline water are expected to differ in mineral content, physical characteristics, and chemical composition compared to DSW. Accurately identifying and comparing these differences can confirm whether the characteristics or effects observed in DSW are indeed unique. Utilizing diverse sample types to validate the attributes or impacts of DSW allows for a more extensive and meaningful interpretation of study results.

In summary, mineral-rich DSW-derived mineral water, specifically HMLS, holds promise as a functional food with a variety of health benefits, including notable anti-inflammatory and anti-ulcer properties. Although this current investigation has been confined to animal models, its findings serve as a foundation for subsequent clinical trials that are crucial for substantiating the positive effects attributed to the consumption of HMLS on human gastric health.

## 4. Materials and Methods

### 4.1. Preparation of DSW-Derived Mineral Water and Quantitative Analysis of Minerals in Samples

The DSW samples were prepared at the Sampio Fermentation Research Center (Cheongju, Republic of Korea). The DSW was derived from a depth of 510 m in the East Sea, and the DSW was prepared by reverse osmosis and electrodialysis using a tabletop electrodialysis device. The DSW samples were produced in two categories: trace mineral (TM) and high magnesium low sodium (HMLS). For the quantitative analysis of minerals in the DSW and DSW-derived mineral waters, each sample was injected in an inductively coupled plasma atomic emission spectrometer (ICP–AES) under conditions described in a previous study [19].

### 4.2. Animals

Twenty-four (male, 7 weeks old) Sprague Dawley rats (SD rats) were purchased from Orient Bio (Gapyeong, Republic of Korea). The animals were allowed ad libitum access to diet and water for 3 weeks and were housed at a temperature of 21 ± 2 °C with a 12-h light and dark cycle. The protocol was approved by the Animal Experimental Ethics Committee of KPC (Ethics no. P193040).

### 4.3. Indomethacin-Induced Gastric Ulcer Model

Animals were randomly divided into four groups (*n* = 6 rats/group): group 1, normal control group (CON); group 2, indomethacin-only group (IND); group 3, indomethacin with TM group (TM); and group 4, indomethacin with HMLS group (HMLS). The CON group and the IND group were allowed to ingest tap water irradiated with ultraviolet rays and filtered through an oil sterilizing filter for 3 weeks, whereas the TM and HMLS groups were provided with mineral water ad libitum for 3 weeks.

Gastric ulcers were induced with indomethacin (30 mg/10 mL/in 0.5% CMC solution) on the last day of the experiment, and the normal control group was administered the same amount of 0.5% CMC solution.

### 4.4. Histological Analysis

For the histopathological analysis conducted at Labcore (Seoul, Republic of Korea), 10% formalin-fixed gastric tissue segments were embedded in the paraffin and sectioned into 4 μm slices. These sections were stained with hematoxylin and eosin (H&E), and Alcian blue-periodic acid Schiff (AB-PAS) staining. Histopathological changes were assessed using a microscope (Olympus, Tokyo, Japan) at 100-fold magnification.

### 4.5. Measurement of Gastric Inflammatory Markers

Myeloperoxidase (MPO) activity and the concentrations of cyclooxygenase-1 (COX-1), COX-2, prostaglandin E_2_ (PGE_2_), TNF-α, and interleukin 10 (IL-10) were quantified using commercially available ELISA kits following the manufacturer’s instructions. Tissue samples were initially rinsed with PBS, then homogenized in PBS to obtain the supernatant and diluted. Subsequently, samples and standards were dispensed into each well, and substrate solutions were added after incubation. The TMB substrate was dispensed into each well in the dark, and the absorbance was measured at 450 nm within 5 min after adding the stop solution.

### 4.6. mRNA Expression in Gastric Tissue

Total RNA extraction from the gastric tissue were performed using TRIzol (Life Technologies, Rockville, MD, USA). Subsequently, cDNA synthesis was accomplished using a Transcriptor First Strand cDNA Synthesis Kit (Hoffmann-La Roche Ltd., Basel, Switzerland). RT–qPCR was conducted using a LightCycler 96 system^®^. The calculation of relative mRNA levels was conducted using the comparative 2^−ΔΔCT^ method, with normalization to β-actin levels (Supplementary Table S1).

### 4.7. Total RNA Sequencing

The RNA purity of gastric tissue was determined by analyzing 1 μL of total RNA extract on a NanoDrop 8000 spectrophotometer. Total RNA integrity was verified using an Agilent Technologies 2100 Bioanalyzer with RNA Integrity Number (RIN) values. Total RNA sequencing libraries were prepared in accordance with the manufacturer’s instructions (Illumina TruSeq Stranded Total RNA Sample Preparation Kit with Ribo-zero). Tagged libraries were combined in equimolar amounts following qPCR using KAPA SYBR FAST qPCR Master Mix (Kapa Biosystems, Wilmington, MA, USA). RNA sequencing was performed using an Illumina NovaSeq 6000 system according to the protocol provided for 2 × 100 sequencing. Reads obtained from each sequencing experiment were assembled, and the abundance of transcripts was calculated in fragments per kilobase of transcript per million mapped reads (FPKM) using Cufflinks software (version 2.2.1).

### 4.8. Statistical Analysis

Statistical analyses were conducted using SAS 9.4 (SAS Institute, Cary, NC, USA). All data are presented as the mean ± standard error (S.E.). An analysis of variance (ANOVA) along with the Duncan’s multiple comparison test were used for statistical assessment. A *p*-value of <0.05 was considered indicative of statistical significance.

## 5. Conclusions

In conclusion, high Mg^2+^ DSW-derived mineral water, particularly HMLS, shows promise as a functional food with potential anti-inflammatory and anti-ulcer properties. This study demonstrated that HMLS intake effectively alleviated indomethacin-induced gastric ulcers in a rat model, improving gastric mucosal health and mucin stability and reducing inflammation. These findings suggest that mineral-rich water sources such as HMLS could be a valuable addition to strategies for gastric health, with implications for future clinical trials and human studies to prevent and manage gastric ulcers and related inflammatory conditions.

## Figures and Tables

**Table 1:** Mineral contents of the DSW-derived mineral water sample ^1^.

Sample ^2^	Mineral Contents (ppm) ^3^				Na^+^/Mg^2+^	Mg^2+^/Ca^2+^
	Na^+^	Mg^2+^	K^+^	Ca^2+^		
DSW	10,700	1380	480	390	7.75	3.5
TM	29.5	6.7	1.7	2.4	4.4	2.8
HMLS	36	179	1.7	41.5	0.2	4.3

^1^ The ion contents of the sample prepared by dialysis of deep-sea water are presented. ^2^ DSW, deep-sea water; TM, trace minerals; HMLS, high magnesium low sodium; ^3^ Na^+^, sodium; Mg^2+^, magnesium; K^+^, potassium; Ca^2+^, calcium.

**Table 2:** Effect of DSW-derived mineral water on gastrointestinal tissue weight ^1^.

Group ^2^	Stomach Weight (g/Body Weight)
	Mean ± S.E.
CON	1.30 ± 0.13
IND	1.54 ± 0.13
TM	1.53 ± 0.13
HMLS	1.50 ± 0.09

^1^ Data are presented as the mean ± standard error (S.E.). Changes in stomach tissue weight after autopsy. ^2^ CON, control (*n* = 6); IND, gastric ulcer induced by indomethacin (50 mg/10 mL/kg B.W. in 0.5% CMC solution) (*n* = 6); TM, gastric ulcer induced by indomethacin (50 mg/10 mL/kg B.W. in 0.5% CMC solution) with TM (*n* = 6); HMLS, gastric ulcer induced by indomethacin (50 mg/10 mL/kg B.W. in 0.5% CMC solution) with HMLS (*n* = 6).

**Table 3:** Identification of inflammatory response-related gene expression in the IND vs. HMLS group (FDR < 0.1).

Gene ID	Gene Name	Fold Change	FDR
Downregulated			
Igfbp4	Insulin-like growth factor binding protein 4	−1.087	0.001
Tnfrsf11b	Tumor necrosis factor receptor superfamily member 11b	−1.114	0.001
Serpina1	Serpin family A member 1	−1.150	0.001
C4a	Complement component 4A	−1.206	0.021
Tac1	Tachykinin precursor 1	−1.225	0.013
C4b	Complement component 4B	−1.368	0.166
Gper1	G protein-coupled estrogen receptor 1	−1.484	0.479
Ccl21	C-C motif chemokine ligand 21	−1.613	0.001
Ccl7	C-C motif chemokine ligand 7	−1.724	0.048
Calca	Calcitonin-related polypeptide alpha	−2.199	0.008
Serpina3n	Serine (or cysteine) peptidase inhibitor, clade A, member 3 N	−2.488	0.001
Upregulated			
Tlr5	Toll-like receptor 5	1.200	0.001
Ccl20	C-C motif chemokine ligand 20	1.529	0.001

## Data Availability

The data presented in this study are contained within the article and Supplementary Materials.

## Supplementary Materials

The following supporting information can be downloaded at: https://www.mdpi.com/article/10.3390/ijms242417430/s1.
