# Supplementary material for: Exploring the Effect of Deep-Sea Water on the Therapeutic Potential of the Anti-Inflammatory Response in an Indomethacin-Induced Gastric Ulcer Rat Model
Source: Int J Mol Sci. 2023 Dec 13;24(24):17430. doi: 10.3390/ijms242417430 (PMC10743565; doi:10.3390/ijms242417430)
Supplement: Supplementary file 1 [file ijms-24-17430-s001.zip › ijms-2745360-supplementary.pdf]

International Journal of
Molecular Sciences

Supplementary Table S1. Primers used for RT–qPCR analysis in gastric ulcers

Gene	Forward Primer	Reverse Primer
COX-1	ttctgccctctgtacccaaa	ggccagaagatgaatatctggta
COX-2	tacaccagggcccttcct	tccagaacttcttttgaatcagg
PGES	gcgttgaaacgtggaggt	atcgtctccatgtcgttgc
IFN-γ	cttttcatagtgcctgtggttg	gatggcagtctagcgttcact
TNF-α	tgaacttcggggtgatcg	gggcttgtcacgagttt
IL-1β	tgtgatgaaagacggcacac	cttcttctttgggtattgtttgg
IL-2	ctgcaaaggaaacacagcag	tggggagtttcagattcttgtaat
IL-6	cctggagtttgtgaagaacaact	ggaagttggggtaggaagga
IL-4	ttgctgcctggcaaaatgtc	acagtgcagcttaccaagcc
IL-10	agtggagcaggtgaagaatga	ggccagaagatgaatatctggta
Tnfrsf11b	gaggtttccagaggaccaca	tgtccattcaatgatgtccaa
Ccl20	ggggtactgctggcttacct	ggcagcagtcaaagttgctt
β-actin	cccgcgagtacaaccttct	cgtcatccatggcgaact

Primer sequences. COX-1; cyclooxygenase-1, COX-2; cyclooxygenase-2, PGES; prostaglandin E synthase, IFN-γ; interferon gamma, TNF-α; tumor necrosis factor alpha, IL-1β; interleukin 1 beta, IL-2; interleukin 2, IL-6; interleukin 6, IL-4; interleukin 4, IL-10; interleukin 10, Tnfsf11b; TNF receptor superfamily member 11b, Ccl20; c-c motif ligand 20, β-actin; beta actin.
